# Instruments and Measurement Techniques to Assess Extremely Low-Frequency Electromagnetic Fields

**DOI:** 10.3390/s25154866

**Published:** 2025-08-07

**Authors:** Phoka C. Rathebe, Mota Kholopo

**Affiliations:** Department of Environmental Health, Faculty of Health Sciences, Doornfontein Campus, University of Johannesburg, P.O. Box 524, Johannesburg 2006, South Africa; motaliok@gmail.com

**Keywords:** ELF-EMFs, instrumentation, short-term monitoring, long-term measurements, health and safety standards, SQUID, exposure assessment

## Abstract

This study presents a comprehensive evaluation and selection framework for extremely low-frequency electromagnetic field (ELF-EMF) measurement instruments. Recognizing the diversity of application environments and technical constraints, the framework addresses the challenges of selecting appropriate tools for specific scenarios. It integrates a structured, quantitative approach through a weighted scoring matrix that evaluates instrumentation across six criteria: monitoring duration, sensitivity, environmental adaptability, biological/regulatory relevance, usability, and cost. Complementing this is a logic-based flowchart that visually guides decision-making based on user-defined operational needs. The framework is applied to a realistic occupational case study, demonstrating its effectiveness in producing evidence-based, scenario-sensitive instrument recommendations. This method provides stakeholders with a transparent and adaptable tool for ELF-EMF device selection.

## 1. Introduction

Extremely low-frequency electromagnetic fields (ELF-EMFs) are generally defined as electromagnetic fields with a frequency up to 300 Hz according to the IEEE and ICINRP guidelines, although some sources extend this upper limit to 1 kHz or 3 kHz for practical considerations in industrial contexts [1,2]. Previous definitions extending to 100 kHz are now recognized as encompassing intermediate frequency (IF) fields [3]. They are forms of non-ionizing radiation with insufficient energy to ionize atoms but are capable of influencing biological systems through mechanisms like free radical production and cellular signaling [4]. Although a growing body of research has suggested potential associations between prolonged ELF-EMF exposure and adverse health outcomes, including childhood leukemia, neurodegenerative disorders, and hematological abnormalities, the overall evidence base remains inconclusive and at times contentious. Proposed biological mechanisms such as oxidative stress induction, intracellular calcium disruption, and alterations in gene expression provide plausible pathways for ELF-EMF effects on living systems [4], but these mechanisms have not been consistently demonstrated across experimental conditions. Epidemiological studies often report statistical associations, such as those by Zhang et al. [5] and Surender et al. [6], yet many suffer from methodological limitations including exposure misclassification, recall bias, confounding by other environmental factors, and variability in outcome definitions. Critically, large-scale reviews and meta-analyses, including those cited by ICNIRP and the World Health Organization [3], have highlighted substantial heterogeneity in findings, with several high-quality studies reporting null or equivocal results. These uncertainties have led regulatory agencies to adopt precautionary exposure limits while simultaneously emphasizing the need for higher-quality exposure assessment tools. In this context, the present review does not presuppose the pathogenicity of ELF-EMFs but rather addresses a central bottleneck in the field: the lack of standardized biologically meaningful measurement methodologies that are robust across varied environmental contexts. By improving the precision and contextual relevance of ELF-EMF monitoring, future research may better resolve the ongoing uncertainties surrounding health risk attribution.

Measuring ELF-EMFs is essential across various contexts of occupational exposure, residential environments and environmental monitoring. In occupational exposure, workers in industries using high-power electrical equipment are routinely exposed to ELF-EMFs, necessitating accurate exposure monitoring for health and safety compliance [7]. In residential environments, it is necessary to understand exposure from household appliances and proximity to power lines to help address public concerns and mitigate potential health risks [8]. In environmental monitoring, high-voltage power transmissions and substations are significant environmental sources, necessitating surveillance to ensure compliance with international exposure guidelines [9].

Accurate measurement and assessment of ELF-EMF exposure has become a critical component in environmental health research and occupational safety programs [8,9]. The challenge lies not only in detecting weak and variable electromagnetic fields but also in determining the relevance of such measurements to health outcomes. Historically, measurement strategies have relied on handheld Gauss meters and fixed monitoring systems, primarily yielding short-term, point-in-time exposure assessments, especially in dynamic environments such as workplaces or urban infrastructures. In contrast, recent advances in sensor technologies such as wearable IoT-enabled devices, fiber-optic systems, and quantum-level magnetometers have enabled continuous monitoring over extended periods, supporting long-term exposure assessment and correlation with physiological data [10]. For example, wearable dosimeters like EMDEX Lite and ExpoM-RF have been successfully deployed in shift-length occupational exposure assessments [11,12], while IoT-based ELF meters allow for real-time exposure logging in smart grid environments [6,13]. SQUID systems have also seen renewed application in biometric research due to their femtotesla (femtotesla per root hertz (fT/√Hz)) sensitivity [14,15]. Fiber Bragg grating (FBG)-based sensors further offer distributed sensing over large areas with high spatial fidelity [16,17].

Despite these innovations, there remains a gap in the literature concerning the comparative evaluation of short-term versus long-term measurement techniques, particularly regarding sensitivity, practicality, environmental adaptability, and relevance to health-oriented research. Additionally, inconsistencies in calibration protocols and standardization frameworks contribute to data variability across studies, limiting their regulatory and scientific utility. Prior reviews have often focused on hardware specifications or isolated use cases without evaluating how instrument type and deployment duration affect biological interpretation and public health applications [8,16,18,19]. This review seeks to critically assess and compare existing ELF-EMF measurement instruments and techniques through the lens of their temporal strategy: short-term or long-term. Examining the technical performance, deployment contexts, and biological applicability of each approach, the paper addresses the following central question: *Which measurement approach and instrument is best suited to quantify ELF-EMF exposure across real-world settings?*

In doing so, the review offers a structured decision-making framework for researchers, engineers, and occupational health professionals, and regulatory authorities involved in ELF-EMF surveillance. It further identifies existing limitations and emerging opportunities for the integration of biosensing, AI-powered analytics, and harmonized standards into future ELF-EMF measurement systems. While ELF-EMF instrumentation has been previously reviewed, most of the existing literature has emphasized hardware design or device-specific validation. This study addresses a distinct gap by systematically comparing measurement tools based on their deployment duration (short-term vs. long-term) and contextual suitability for biological regulatory relevance. Instead of providing an exhaustive review of all ELF-EMF studies, this paper synthesizes a set of technically validated, representative instruments to propose a critical decision-making framework. This approach is particularly valuable for stakeholders in public health, occupational safety, and infrastructure who must select measurement strategies that are not only technically accurate but also biologically and operationally meaningful.

## 2. Methodology

This review evaluates the suitability of measurement approaches and instruments used in the quantification of extremely low-frequency electromagnetic fields (ELF-EMFs), particularly concerning their applicability in either short-term (spot measurement) or long-term (continuous monitoring) exposure contexts. The objective was to determine which technique provides more reliable, accurate, and context-sensitive data across occupational, residential, and biomedical environments. A structured literature search was conducted using four major academic databases: IEEE Xplore, ScienceDirect, Scopus, and PubMed, which constituted the complete set of sources used to identify empirical studies relevant to ELF-EMF measurement within the 0–300 Hz range. The exact search terms used were “ELF EMF measurement,” “short-term EMF monitoring,” “long-term EMF dosimetry,” “Gauss meter,” “SQUID magnetometer,” “wearable ELF sensor,” and related keywords. The search spanned publications from January 200 to March 2024, capturing both legacy technologies and recent advancements. The initial search retrieved 142 articles. After removing duplicates and screening titles and abstracts for relevance, 73 articles were selected for full-text review. Applying predefined inclusion criteria—namely, studies providing empirical deployment data, technical specifications, and context-specific applications—38 studies were retained for detailed analysis. Studies were excluded if they focused solely on theoretical modeling, addressed intermediate or higher frequencies, or lacked adequate instrumentation detail. No review management software was used; instead, articles were manually screened and extracted by both authors. A standardized data extraction matrix was employed to document key parameters from each study, including instrument type, operating principle, frequency range, sensitivity, deployment context (e.g., occupational, residential, biomedical), duration (short-term or long-term), and calibration methods. The relevance of each instrument to biological outcomes research was then considered in the interpretation of its practical suitability. Discrepancies in categorization or interpretation were resolved through author consensus.

**Figure 1:**
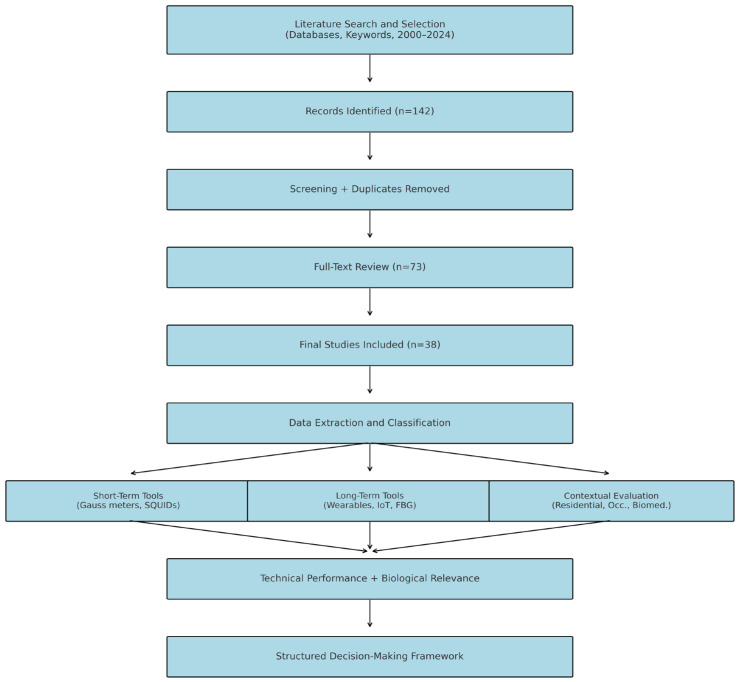
Schematic representation of the review of the methodology and instrument comparison framework.

This review was designed as a targeted comparative synthesis rather than a systematic review, with the primary aim of evaluating the practical suitability of ELF-EMF measurement instruments in either short-term or long-term (continuous) deployment contexts. The selection of studies was therefore guided not by exhaustiveness but by instrumental representativeness, regulatory relevance, and validation in real-world or occupational applications. Specifically, we prioritized peer-reviewed sources that (i) described ELF-EMF measurement within the 0–300 Hz range, (ii) provided clear operational specifications or deployment strategies, and (iii) offered insight into the instrument’s performance, sensitivity, or biological/occupational application. Studies without empirical deployment data, unclear instrument design, or limited relevance to the stated frequency range were excluded to ensure comparability across reviewed technologies. In this study, we acknowledge that the number of studies does not reflect the full scope of the ELF-EMF measurement literature. However, the selected sources represent a diverse and strategically curated subset of validated technologies, sufficient to support a comparative work. This framework is intended to assist researchers, engineers, and regulatory stakeholders in selecting instruments based on temporal monitoring needs, biological relevance, and environmental context. Future work may extend this framework through formal meta-analyses or systematic reviews that quantify field performance across broader geographic, demographic, and technical variations. For the present review, our goal is to provide a conceptually strong, application-driven lens through which the utility of ELF-EMF instruments can be interpreted and improved. A schematic of the review methodology and comparative framework is presented in Figure 1 to illustrate the study’s selection logic evaluation process.

## 3. Understanding ELF-EMFs: Sources and Physical Properties

ELF-EMFs are a form of non-ionizing radiation within the frequency range of 0 to 300 Hz, most commonly generated by power systems operating at frequencies of 50 to 60 Hz. These fields are prevalent in modern environments due to the widespread use of electrical infrastructure and devices. ELF-EMFs are generated by various sources, including electrical power lines, household appliances, and industrial equipment. These fields are of particular concern in occupational and residential settings, where long-term exposure can occur. Due to the nature of ELF-EMFs, they are often subject to continuous monitoring and regulation to ensure public and occupational safety. Measurement of ELF-EMFs is based on three fundamental physical parameters. The electric field strength (E), expressed in volts per meter (V/m), quantifies the intensity of the electric field created by voltage. Magnetic flux density (B), typically measured in microtesla (µT) or milligauss (mG), reflects the strength of the magnetic field generated by the electric current. Finally, frequency is measured in hertz (Hz) and represents the rate at which the electric and magnetic fields oscillate. These parameters form the basis for all ELF-EMF assessments and understanding them is crucial in determining potential exposure risks and in the development of measurement and regulation protocols [8,20,21]. Electric field strength (E) is calculated as E = V/d, where V is the electric potential and d is the distance between the points. This equation governs how voltage in transmission lines or appliances translates to localized field intensity. In realistic scenarios, especially near overhead power lines, electric field values may reach several kV/m depending on the load and configuration [8,22].

### 3.1. Common Sources of ELF-EMFs

ELF-EMFs originate from both natural and man-made sources. Natural sources of ELF-EMFs include the Earth’s magnetic field and atmospheric phenomena such as lightning. These natural occurrences contribute to the background ELF-EMF levels that exist in the environment. While the Earth’s magnetic field is relatively constant, lightning and other atmospheric events can cause brief spikes in ELF-EMF exposure. On the other hand, man-made sources are more prevalent and can contribute to higher exposure levels, particularly in industrial and residential environments. These sources include power transmission infrastructure, such as high-voltage transmission lines and substations, which generate significant ELF-EMFs. Industrial equipment, including electrical furnaces, motors, and welding devices, also emit ELF-EMFs during operation. Additionally, household electrical appliances and electric transportation systems like electric trains and vehicles are common contributors to ELF-EMF exposure in everyday life [8].

### 3.2. Exposure Level and Variability

In residential areas, magnetic flux densities from ELF-EMFs typically remain below 0.5 µT, while occupational settings such as power plants or semiconductor manufacturing can experience levels exceeding 1.5 µT [6]. Recent field studies provided more exposure data across diverse environments. For example, Tampouratis et al. [21] reported flux densities ranging from 0.05 to 1.8 µT in industrial environments using IoT-enabled meters, while Surender et al. [6] recorded average exposures of 1.2–2.1 µT among thermal power plant workers. These statistics illustrate the variability of exposure and the importance of continuous monitoring in dynamic occupational settings. Factors include proximity to the sources of the fields and the current load passing through the electrical systems. These variations necessitate robust monitoring to assess potential health impacts and ensure compliance with international safety standards.

Faraday’s Law and induction relevance:

In dynamic ELF environments, the relationship between a time-varying magnetic field and induced electric fields is governed by Faraday’s Law:

(1) $$E=-\frac{d\Phi_{B}}{dt}$$

where ε  is the electromotive force (v) and $\Phi_{B}$ is magnetic flux. Magnetic flux is defined as:

(2) $$\Phi_{B}=B\cdot A\cdot \cos\left(\theta \right)$$

where B is the magnetic flux density (T), A the perpendicular area (m^2^), and θ the angle of incidence. The relationship is foundational for understanding how electric fields are induced in biological systems exposed to ELF-EMFs [4,23,24].

Experimental studies confirm that the induced electric field, not just the magnetic field itself, is the primary driver of biological effects, such as intracellular calcium signaling and gene activation [25]. Recent research has expanded our understanding of the biological mechanisms through which ELF-EMFs exert effects. Studies have shown that low-frequency fields can modulate oxidative stress pathways [26], influence calcium efflux and intracellular signaling [27], and alter gene expression in neural and reproductive tissues [28,29]. These findings support the need for measurement systems that not only quantify field strength but also characterize parameters such as frequency and waveform complexity that are biologically consequential. The theoretical basis and linear flux–EMF relationships have been reaffirmed in modern experimental setups [27,30] and theoretical reviews [31].

Skin depth equation for frequency dependence:

The penetration of ELF fields is characterized by skin depth:

(3) $$\delta =\sqrt{\frac{2}{\mu \sigma \omega }}$$

δ=  where μ  is permeability, σ is conductivity, and ω=2πf represents regular frequencies. This depth can be several centimeters, making ELF-EMFs uniquely capable of penetrating into biological tissues compared to higher-frequency radiation [32].

Waveform complexity and harmonics:

Real-world ELF-EMFs rarely exhibit ideal sinusoidal waveforms. Harmonic distortion caused by electronic devices introduces frequencies that are integer multiples of the fundamental (e.g., 150 Hz, 250 Hz) [33]. This is quantified using Total Harmonic Distortion (THD):

(4) $$\mathrm{THD}=\frac{\sqrt{\sum_{n=2}^{\infty }V_{n}^{2}}}{V_{1}}$$

where $V_{n}$ are RMS voltages of harmonics and $V_{1}\;$ is the fundamental component [34]. Higher THD implies greater biological interaction potential due to field irregularity and pulsed peaks [35]. Understanding ELF-EMF exposure requires more than assessing magnitude alone; waveform complexity, harmonic distortion, and exposure duration all significantly influence biological responses. From examples, Touitou et al. [12] demonstrated that rotating-shift workers exposed to non-sinusoidal ELF-EMFs experienced disrupted melatonin rhythms and sleep quality, while Chen et al. [36] identified mitochondrial dysfunction linked to irregular field patterns. These findings highlight that biologically meaningful exposure assessments must account for waveform shape, not just intensity or frequency.

## 4. Principles and Comparative Evaluation of ELF-EMF Measurement Instruments

Accurate quantification of extremely low-frequency electromagnetic fields (ELF-EMFs) depends on understanding the physical characteristics of these fields and the operation of instruments designed to detect them. This section expands on the principles behind leading measurement instruments and presents a comprehensive comparative analysis based on technical performance, operational suitability, sensitivity, and application contexts.

### 4.1. Fundamental Parameters Measured

The accurate assessment of ELF-EMFs requires precise measurement of several interrelated physical parameters. These include (i) electric field strength, (ii) magnetic field flux density, (iii) frequency, and in specific contexts, (iv) waveform characteristics. Each parameter contributes to understanding both the presence and potential impact of ELF-EMFs in environmental and occupational settings. Electric field strength is typically measured in volts per meter (V/m) and reflects the force exerted by the field on a unit charge. It provides insight into the spatial distribution of electric potential in an environment, especially in proximity to energized equipment or transmission lines. While electric field measurements are particularly relevant in high-voltage environments, magnetic field parameters are more commonly used for health risk assessments due to their stronger penetration in biological tissues and their correlation with current flow. Magnetic flux density, represented in microteslas (µT) or milligauss (mG), is the most widely measured quantity in ELF-EMF monitoring and is often used as the reference metric in exposure guidelines. This parameter quantifies the intensity of the magnetic field generated by alternating current in power systems and electrical devices. It directly influences the potential for interaction with biological systems, such as through induced currents in tissues or modulation of cellular activity. Numerous epidemiological studies have linked chronic exposure to magnetic fields above certain thresholds to increased health risks, including childhood leukemia and neurodegenerative disorders, which has led to international guideline limits of 100 µT for public exposure and 500 µT for occupational settings as recommended by ICNIRP and reaffirmed in the later literature [1,8].

Frequency, measured in hertz (Hz), indicates the rate at which the electric and magnetic fields oscillate. ELF-EMFs are defined in the range from 0 to 300 Hz, although practical applications in power transmission focus on 50 Hz or 60 Hz, depending on regional infrastructure. The biological significance of frequency stems from the resonance properties of cellular components and tissues, which may exhibit increased sensitivity to specific frequency bands. Additionally, distinguishing ELF from intermediate-frequency fields (300 Hz to 10 kHz) is critical in standardizing measurement protocols and interpreting exposure outcomes [3,5]. Another important consideration in ELF-EMF measurement is the waveform of the field, particularly whether it is sinusoidal, pulsed, or contains harmonics and transient components. While most regulatory assessments assume sinusoidal waveforms from alternating current sources, real-world fields often include non-sinusoidal fluctuations caused by switching devices, industrial machinery, and transient fault events. These waveform characteristics are particularly relevant in biomedical research, as they may interact differently with biological systems compared with smooth, continuous fields [4,36]. Recent research has emphasized the biological relevance of ELF-EMF waveform complexity. For example, various waveforms such as sinusoidal, triangular, and pulsed forms have been shown to differently affect cell morphology and proliferation in human osteosarcoma cells, with fractionated exposures leading to measurable cytoskeletal remodeling [28]. Likewise, using Fourier-transform infrared spectroscopy, Li et al. [37] identified spectral shifts in rat brain and testis tissue after 20-day ELF-EMF exposure, highlighting frequency and amplitude-specific molecular alterations. These findings support the necessity of characterizing not only field strength but also waveform and harmonic content in ELF-EMF exposure assessments.

Recent studies emphasized the need to address field irregularities. Paniagua et al. and Schuderer et al. [38,39] demonstrated that harmonic distortion in power line signals can increase neurobiological activity in vitro, suggesting that waveform content beyond RMS values must be regularly measured. Most studies advocated for the inclusion of spectral characteristics in ELF-EMF environmental assessments due to their predictive utility in modeling oxidative stress and endocrine disruption [26,40,41].

### 4.2. Instrumentation Summary: Practical Principles for Application Contexts

The accurate and meaningful measurement of ELF-EMFs depends not only on sensitivity and physical design but also on the adaptability of instruments to diverse environments, their deployment duration, and their ability to yield biological or regulatory relevant data. This section presents a comprehensive review of the major ELF-EMF measurement instruments in use today, with a particular focus on their practical applicability, operational strengths and weaknesses, and alignment with current exposure assessment goals. Emphasis is placed on recent technological developments, validated deployment contexts, and the suitability of each instrument type for short-term or long-term monitoring.

#### 4.2.1. Magnetometers and Gauss Meters

Hall-effect magnetoresistive Gauss meters are foundational tools in ELF-EMF measurement. These devices are widely used for short-term spot measurements in residential, workplace, and laboratory settings [42,43,44]. They operate by converting magnetic flux density into a voltage or resistance signal using Lorentz force or magnetoresistive principles [45]. Their portability, affordability, and user-friendliness make them a standard for initial assessments and electromagnetic compliance checks [46]. Recent studies have demonstrated the increasing sophistication of these tools. Microelectromechanical system (MEMS)-based low-noise variants offer sensitivity in the sub-nanotesla range, expanding their use in indoor environments and precision mapping [47]. However, it is important to note that such nanotesla resolutions far exceed the requirement for regulatory compliance assessments, which typically rely on detecting magnetic densities in the microtesla range. For compliance purposes, instruments with sensitivities between 0.1 µT and 100 µT are generally sufficient as prescribed by ICNIRP and IEEE standards. Ultra-sensitive instruments are more relevant in experimental, biomedical, or diagnostic contexts where weak fields are of interest. Gauss meters have been used in a variety of applications, from residential proximity assessment to power lines to field mapping in classrooms and public buildings [43,48,49]. Despite these benefits, these instruments are limited in their inability to capture temporal variations, making them unsuitable for long-term monitoring. They are also prone to measurement distortion in high EMI environments, as confirmed in studies evaluating ELF noise from incubators, routers, and power systems in laboratory environments [50]. To address these shortcomings, researchers have developed shielding protocols and advanced scanning setups to improve spatial fidelity [51,52]. Nevertheless, Gauss meters remain best suited for fixed-location audits, baseline scans, and diagnostic use cases that do not require time-resolved exposure data.

#### 4.2.2. SQUID Magnetometers

Superconducting Quantum Interference Devices (SQUIDs) are regarded as the most sensitive instruments for ELF-EMF measurement, capable of detecting fields with spectral sensitivities as low as a few femtotesla per root hertz (fT/√Hz). Their extraordinary precision makes them indispensable in controlled biomedical applications, particularly in magnetoencephalography (MEG), magnetoneurography, and magnetocardiography. SQUIDs have long been employed in neuroimaging and cardiac electrophysiology, and their sensitivity is critical for capturing weak biomagnetic fields that conventional sensors cannot detect [14,53]. Recent advances in high-temperature superconducting materials have improved the practicality of SQUIDs. Devices operating at 77 K (liquid nitrogen temperatures) have made SQUID deployment more accessible beyond helium-cooled labs [54,55]. Applications now extend to portable biomagnetic arrays and neurofunctional imaging in spinal cord research [56]. Additionally, innovations such as flux-locked loop (FLL) configurations have enhanced linearity and reduced drift during short-term experiments [15]. Despite these advancements, SQUIDs remain limited by their high cost, complex cryogenics, and the need for electromagnetic shielding. Consequently, they are impractical for long-duration monitoring or field deployment in uncontrolled settings. Their value lies in providing ultra-high-resolution data for short-term, high-stakes biomedical research or experimental studies.

#### 4.2.3. Wearable ELF-EMF Sensors

Wearable ELF-EMF sensors have transformed exposure assessment practices by enabling real-time tracking of magnetic field exposure through repeated sampling at regular intervals, which approximates continuous monitoring over extended durations. These devices are designed for long-term use, typically over hours or full work shifts, and integrate tri-axial magnetoresistive sensors with inertial measurement units, data logging, and wireless telemetry [57,58]. The practicality of wearable devices is particularly evident in occupational exposure monitoring. Studies using tools such as EMDEX Lite and ExpoM-RF have demonstrated successful deployment in electrical utility workers and industrial staff, capturing time-weighted exposure values and exposure peaks that are otherwise missed in spot measurements [59]. Several studies have correlated wearable-based exposure data with biological outcomes, such as reduced natural killer cell activity and altered melatonin rhythms in chronically exposed individuals [12,29,60]. While wearable sensors allow for detailed temporal profiling, they also present challenges. Devices often require frequent recharging and recalibration. Their limited dynamic range (~±100 µT) restricts their use in high-current environments, and motion-induced artifacts may compromise signal accuracy without proper correction algorithms. Nonetheless, recent innovations in edge computing and onboard signal processing, including FFT capabilities, are addressing these limitations [61]. Wearable ELF-EMF sensors have become central to long-term exposure assessment, especially in occupational health and epidemiological research. Devices like EMDEX Lite and ExpoM-FR enable real-time, tri-axial magnetic field monitoring across full work shifts, capturing temporal exposure patterns missed by spot measurements. Beyond their technical utility, these devices have demonstrated biological relevance; studies have linked recorded ELF-EMF exposure to disrupted melatonin rhythms, immune modulation, and circadian misalignment in shift workers [12,60]. Recent advancements integrate motion correction, FFT processing, and physiological telemetry (e.g., heart rate, sleep), supporting multi-modal health studies. While limited by battery life and dynamic range (±100 µT), wearables offer unmatched utility for continuous, biologically contextualized exposure profiling in real-world settings. Wearables increasingly support health-integrated exposure studies, offering opportunities for coupling field data with physiological parameters such as heart rate, cortisol levels, or sleep quality [62].

#### 4.2.4. IoT-Enabled ELF-EMF Meters

IoT-enabled ELF-EMF meters represent a new frontier in scalable distribution monitoring. These systems integrate magnetoresistive sensors with wireless communication modules, enabling networked measurement of field exposure across multiple locations. Deployed in industrial, energy, and urban settings, they offer real-time data acquisition, cloud storage, and automatic anomaly detection. Recent research has applied IoT sensors in smart grid environments to monitor ELF-EMF exposure in proximity to substations and distribution nodes [21]. These meters are also being tested for long-term environmental surveillance in urban planning projects and public health tracking systems. Because they are permanently installed, they eliminate the need for human wearers, allowing longitudinal field capture over months or years. However, IoT systems are not without challenges. They depend on a consistent power supply, reliable data connectivity, and robust data encryption protocols [63]. Additionally, their sensors must be periodically recalibrated, and urban EMI can affect signal fidelity [64]. Despite these challenges, their scalability and automation potential make them ideal for large-scale infrastructure monitoring and population-level exposure modeling.

#### 4.2.5. Fiber-Optic Magnetometers

Fiber-optic magnetometers, especially those based on fiber Bragg gratings (FBGs) coated with magnetostrictive materials, offer an elegant solution to ELF-EMF detection in environments where EMI immunity and spatial scalability are critical. These systems detect magnetic field-induced strain as wavelength shifts in the fiber, allowing for long-range, distributed field monitoring. FG sensors have been extensively used in high-voltage environments such as power substations, where metallic sensors may suffer from interference or risk of failure [65]. In smart infrastructure applications, phase-sensitive optical time-domain reflectometry (φ-OTDR) allows kilometers of sensing fiber to be monitored with high spatial resolution [17,66]. Dual-FBG and temperature-compensated designs have further enhanced accuracy by reducing thermal drift and environmental artifacts [67]. Although powerful, fiber-optic systems require precise AC calibration and are costlier than electronic sensors. Installation requires technical expertise, and the interpretation of spectral data is computationally intensive. Nonetheless, their EMI immunity, scalability, and non-electronic nature make them ideal for use in MRI rooms, smart grids, and areas with stringent electromagnetic compatibility requirements. Fiber-optic ELF-EMF sensors, particularly FBG-based systems, provide high resolution and EMI-immune monitoring, making them ideal for electrically sensitive environments such as MRI suites, neonatal ICUs, and high-voltage substations. These sensors convert magnetic fields induced by strain into spectral shifts, enabling distributed multi-kilometer monitoring with minimal interference. Deployed in smart grids and critical care units, they allow for long-term surveillance without electrical components, which is crucial where standard sensors fail [65]. Though they are calibration-intensive and costly, their precision, spatial scalability, and immunity to ambient noise make them indispensable for infrastructure monitoring and emerging public health applications where electronic interference cannot be tolerated.

### 4.3. Technical and Environmental Challenges

Despite ongoing innovations, there are key technical challenges in EMF measurement; these include interference from environmental sources, sensor sensitivity and calibration, field homogeneity, and safety and shielding. Several factors influence the accuracy and reliability of ELF-EMF measurements. First, interference from environmental sources such as ambient electromagnetic noise from devices like mobile phones and Wi-Fi networks can distort readings, with high voltage power lines and urban environments further exacerbating this issue [68]. Second, sensor sensitivity and calibration play a crucial role; ensuring that sensors are sensitive enough to detect low-intensity ELF-EMFs without being saturated by stronger fields is essential for maintaining accurate measurements. Regular calibration is necessary to ensure equipment continues to function properly over time [69]. Third, field homogeneity presents a challenge, particularly in laboratory settings where uniformity is critical for obtaining reproducible results [15]. Lastly, low-frequency noise and transients, particularly those from intermittent or near DC ELF components (<1 Hz), complicate comparisons. Techniques like amplitude modulation and signal averaging, as demonstrated in Rydberg atom-based sensors, show promise in overcoming this limitation [10]. In addition to these challenges, the quantification of measurement uncertainty remains a critical yet often underreported aspect of ELF-EMF assessment. Measurement uncertainty remains a challenge in ELF-EMF exposure assessment, yet few studies adequately address its qualification. This review identifies key sources of uncertainty, including sensor drift, electromagnetic interference, positional variability, and sampling resolution. Calibration errors, particularly in high-sensitivity instruments like SQUIDs and fiber-optic magnetometers, can significantly skew data if not corrected using standard traceable methods such as Helmholtz coils [70]. Environmental noise, especially in urban and industrial settings, introduces distortion that is difficult to isolate without spectral filtering or shielding [68]. Orientation errors in wearable and handheld devices further compromise spatial accuracy, while limited rates in long-term monitors can misrepresent transient peaks. These factors collectively contribute to typical measurement uncertainty margins of ±5% to ±20%, as reported in prior development studies and international standards [59]. In line with ICNIRP and IEEE protocols, this review underscores the need for systematic uncertainty quantification and recommends incorporating expanded uncertainty estimates when interpreting exposure levels against biological thresholds.

### 4.4. Standardization and Measurement Protocols

Measurement standards established by the IEEE and ICNIRP prescribe sensor types, calibration intervals, sampling protocols and exposure thresholds. These guidelines ensure that ELF-EMF assessments are comparable across time, locations and studies. Meaningful characterization requires both short-term spot measurements and long-term continuous monitoring, particularly in occupational and public infrastructure settings [49]. While calibration standards such as those from the ICNIRP and IEEE provide a foundation, true international harmonization remains limited by fragmented regulatory frameworks, proprietary instrumentation, and variable resource capacities. To overcome these barriers, this paper proposes the creation of a global consortium to develop open-source calibration libraries, universal protocol templates, and tiered compliance benchmarks adaptable across contexts. Harmonization should prioritize interoperability, cost-effective validation tools, and standardized reporting formats to enable cross-study comparability and support global public health surveillance. Collaborative alignment among scientific, regulatory, and industrial stakeholders is essential to advance biologically meaningful, internally consistent ELF-EMF monitoring.

### 4.5. Summary of Instrument Comparison

To synthesize the comparative performance of the reviewed ELF-EMF measurement instruments, Table 1 presents a structured summary highlighting their key operating principles, sensitivity ranges, technical advantages, inherent limitations, and ideal application contexts. This comparative framework aims to aid researchers and practitioners in selecting the most appropriate instrumentation based on specific field requirements, measurement goals, and operational constraints.

This comparative summary reflects both the technical evolution of ELF-EMF measurement instruments and their suitability in application-dependent contexts. Recent studies have reinforced the importance of selecting devices based on sensitivity and range, but also biological impact relevance and real-world calibration feasibility.

### 4.6. Supplemental Quantitative Performance Comparison

To complement the qualitative evaluation presented in Section 4.5, Table 2 below offers a quantitative summary of key performance metrics for ELF-EMF measurements. These include typical sensitivity, ranges, accuracy, sampling rates, power requirements, and estimated costs—factors critical for selecting devices in real-world scenarios. The table bridges the gap between theoretical design and operational feasibility, allowing stakeholders to align instrument choice with technical demands, budget constraints, and regulatory standards.

## 5. Comparative Assessment of ELF-EMF Measurement Instruments

This section presents a comparative assessment of ELF-EMF measurement instruments with particular emphasis on their operational context, sensitivity, and suitability for either short-term or long-term monitoring. The instruments are evaluated based on findings from selected peer-reviewed studies and technical reports. The results are organized into three key subsections: (i) comparative summary of study characteristics, (ii) performance evaluation by measurement duration, and (iii) contextual suitability across residential, occupational, and biomedical applications.

### 5.1. Study Characteristics and Instrument Classification

Table 3 below summarizes the key characteristics of selected studies, highlighting the instrument used, measurement context, sensitivity, technique duration (short-term or long-term), and notable strengths and limitations. The aim is to identify trends in performance and deployment based on the instrument type and intended use-case environment.

The limitations listed in Table 3 are further supported by deployment studies. For example, Valič et al. [59] reported battery and calibration issues in wearable dosimeters, Tampouratzis et al. [21] noted power and thermal drift challenges in IoT meters, and Liu et al. [98] observed EMI distortion in handheld Gauss meters. SQUID magnetometers, while highly sensitive, are restricted by shielding and cooling needs [54].

### 5.2. Performance Duration: Short-Term vs. Long-Term Measurement Instruments

Short-term instruments (as detailed in Section 4) are suited for diagnostic and laboratory uses but are less effective in capturing real-word temporal exposure variability. In contrast, long-term measurement systems, including wearable dosimeters and fiber-optic networks, are better equipped for continuous exposure tracking in occupational monitoring scenarios. Wearable devices (see Section 4) enable repeated sampling at a certain rate and are widely used in occupational exposure assessments. Fiber Bragg grating (FBG) sensors with magnetostrictive coating provide high spatial resolution and immunity to electromagnetic interference, making them appropriate for monitoring substations and high-voltage infrastructure [65,66]. Wearable IoT systems represent a growing area in long-term monitoring, with recent studies integrating edge computing and cloud analytics for real-time exposure profiling [21]. However, these systems face challenges related to power supply, thermal drift, and calibration stability.

### 5.3. Environmental Suitability and Use-Case Alignment

The practical application of these instruments depends heavily on the monitoring environment. In residential surveys, low-cost handheld meters are typically sufficient, though care must be taken to mitigate spatial artifacts and EMI [98]. Occupational settings demand wearable solutions with real-time data transmission and motion compensation capabilities, particularly where personnel move between varying EMF zones during a work shift [59]. In biomedical research, high-resolution short-term tools like SQUIDs remain the gold standard for correlating EMF exposure with neurophysiological signals. Recent advances in cryogenic-free high-Tc SQUIDs have improved their viability for experimental labs [54,56]. Lastly, infrastructure and environmental monitoring increasingly relies on long-term optical or IoT-based tools capable of operating under high-EMI conditions with minimal maintenance. These applications, unlike occupational monitoring methods which often emphasize personal-level time-weighted exposure, typically require remote monitoring and near-real-time data transmission to support centralization oversight, rapid anomaly detection, and infrastructure safety compliance. IoT-based ELF-EMF meters in particular are well-suited to this role due to their wireless networking, cloud integration, and automated logging features. These include Φ-OTDR systems and smart grid-integrated sensors [21,66].

### 5.4. Proposed Decision-Making Framework: Structure and Rationale

This study introduces a quantitative, scenario-driven decision-making framework to support the selection of ELF-EMF measurement instruments. The framework evaluates candidate devices against six critical criteria: monitoring duration, sensitivity and accuracy, environmental adaptability, regulatory or biological relevance, usability, and cost. Each criterion is assigned a default weight based on its general importance in field and laboratory contexts (Table 4). The framework is flexible, allowing users to adjust these weights to reflect specific needs or constraints.

### 5.5. Scoring System and Customizable Evaluation

Each instrument is scored from 1 (poor) to 5 (excellent) for all six criteria. A weighted sum of scores determines the overall suitability of each device for a specific use case:

Weighted score = ∑ (Criterion Score × Criterion Weight)(5)

Table 5 presents an example scoring matrix, followed by a final weighted score (Table 6) based on current technology performance.

This model can be easily integrated into a spreadsheet or decision-support tool to enable real-time customization and ranking based on user-defined priorities.

### 5.6. Visual Decision Logic: Flowchart for Instrument Selection

**Figure 2:**
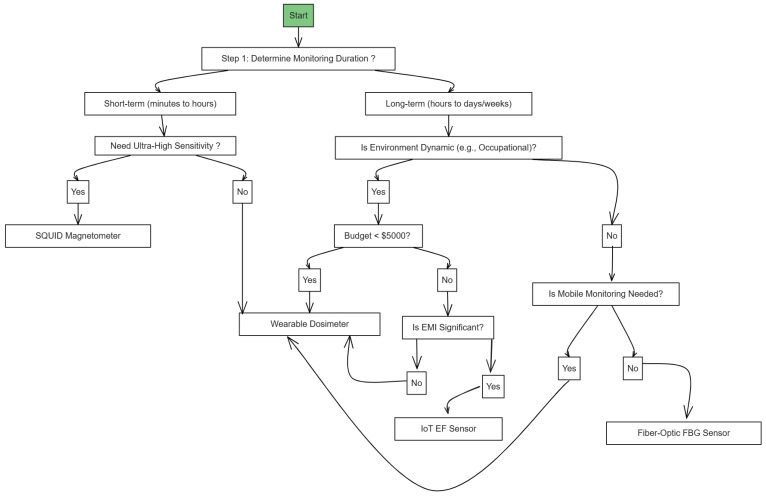
Logic-based decision tree for ELF-EMF instrumentation selection.

Figure 2 presents a decision tree model to operationalize the scoring system. This logic model begins with the monitoring duration and follows conditional branches based on environmental, technical, and financial factors.

The flowchart guides users through a structured evaluation of monitoring duration, sensitivity needs, environmental dynamics, budget limitations, EMI considerations, and mobility requirements. Final recommendations include suitable devices such as SQUID magnetometers, wearable dosimeters, IoT ELF sensors, and fiber-optic FBG sensors based on practical constraints and performance priorities.

Figure 2 presents a visual decision-making flowchart that captures the core logic of the instrument selection process. It begins by evaluating the required monitoring duration (short-term vs. long-term). For short-term needs, a decision on sensitivity guides the choice between a SQUID magnetometer or a wearable dosimeter. For long-term applications, the framework considers whether the monitoring environment is dynamic, such as in occupational or mobile scenarios. Budget constraints, EMI significance, and mobility needs are then used as progressive filters to narrow down options. The resulting recommendations, such as wearable dosimeters, IoT ELF sensors, or fiber-optic FBG sensors, align with both technical requirements and operational feasibility.

### 5.7. Case Study: Substation Worker Exposure Monitoring

A representative scenario involving occupational ELF-EMF exposure monitoring in an industrial setting is presented to demonstrate the practical application of the proposed framework. A regional electricity provider is tasked with assessing the exposure levels experienced by maintenance personnel operating within high-voltage substations. These environments are characterized by persistent electromagnetic fields, intermittent spikes in intensity, and complex interference from heavy-duty electrical equipment. The objective is to identify the most suitable measurement instrument for this case, considering a series of real-world constraints. The monitoring must be continuous over a full work shift, typically 8 to 12 h, thus eliminating devices that cannot support long-duration data logging or battery life. The measurement environment includes high electromagnetic interference (EMI), which further narrows the range of appropriate technologies to those designed with robust shielding or immune mechanisms. Due to the agency’s limited procurement budget, which is less than USD 5000 per unit, certain high-precision systems such as SQUID magnetometers and advanced fiber-optic platforms are excluded solely based on cost. Additionally, any selected device must output data compatible with standards set by ICNIRP and other occupational safety organizations. These outputs should support meaningful analysis using root mean square (RMS) values and time-weighted average (TWA) metrics to meet exposure classification requirements. Applying this logic-driven decision framework described in previous sections, the first filter eliminates instruments that cannot be used long-term, such as traditional handheld Gauss meters or laboratory-only SQUID systems. Next, environmental constraints prioritize solutions that perform reliably in EMI-heavy conditions, favoring fiber-optic sensors or modern IoT-enabled devices. Budget constraints then exclude the fiber-optic options. Of the remaining candidates, wearable ELF dosimeters and IoT ELF sensors remain.

To fully apply the decision-making framework, the evaluation criteria were customized for this specific context. In this scenario, the most critical factors, monitoring duration and environmental adaptability, were given higher weights due to the operational requirements of long-shift monitoring in EMI-prone environments. The adjusted weights for each criterion were as follows (Table 7):

Each candidate device was then scored on a scale of 1 (poor) to 5 (excellent) based on performance within this specific case (Table 8 and Table 9):

Using the weighted sum method:

Wearable ELF Dosimeter: (5 × 0.25) + (4 × 0.15) + (4 × 0.20) + (5 × 0.15) + (4 × 0.10) = 4.45

IoT-Enabled ELF Sensor: (5 × 0.25) + (3 × 0.15) + (4 × 0.20) + (5 × 0.15) + (4 × 0.10) = 3.90

Given these results, the Wearable ELF Dosimeter (e.g., EMDEX Lite) becomes the most suitable solution. It not only aligns with the technical and operational requirements of substation monitoring but also ranks highest when evaluated through the customized decision framework. This outcome is reinforced by the scoring matrix presented in Table 6 and corroborated by prior field studies, validating the EMDEX Lite as both a scientifically justified and operationally practical choice for substation worker exposure assessment [59].

## 6. Discussion

The comparison drawn from the above sections illustrates the diversity and specialization of ELF-EMF measurement instruments, with significant differences in sensitivity, deployment flexibility, and application scope. The central question guiding this review—*Which measurement approach and instrument is best suited to quantify ELF-EMF?*—requires a meticulous consideration of both short-term and long-term methodologies within their practical and regulatory contexts.

### 6.1. Strengths and Weaknesses of Short-Term Instruments

Short-term instruments, notably handheld Gauss meters and SQUID magnetometers, offer critical advantages in terms of portability, immediate deployment, and high sensitivity. Hall-effect and tunneling magnetoresistive (TMR) Gauss meters remain necessary for quick residential or compliance spot checks due to their compact design, sub-nanotesla resolution, and user-friendly interference from ambient sources, which can result in data distortion in uncontrolled environments [99]. SQUID systems, particularly those based on high-Tc superconductors, provide unmatched sensitivity in the femtotesla range and have become essential in advanced biomedical applications such as magnetoencephalography (MEG) and magnetoneurography [54,56].

While effective in laboratory settings, their requirement for cryogenic cooling, electromagnetic shielding, and complex calibration procedures limits their field applicability and confines them primarily to experimental or diagnostic use [15]. Table 10 below provides a side-by-side comparison of short-term and long-term measurement approaches based on key performance parameters.

As shown in Table 10, short-term techniques are most appropriate in situations requiring high spatial resolution, rapid scanning, or controlled exposure evaluation, yet they can detect temporal exposure variations that are crucial in occupational or epidemiological settings.

### 6.2. Advantages and Limitations of Long-Term Monitoring

Long-term ELF-EMF monitoring systems, such as wearable dosimeters and fiber-optics sensor arrays, address many of the limitations associated with short-term techniques by enabling continuous, context-aware tracking. Devices like EMDEX lite and ExpoM-RF offer shift-duration monitoring by periodically sampling ELF-EMF exposure (e.g., every few seconds), coupled with motion artifact correction and wireless data logging, yielding a temporally resolved data set suitable for workplace exposure and epidemiological studies [59]. It should be noted that in occupational studies, real-time data transmission is primarily intended for central monitoring and exposure management by researchers or safety personnel, rather than for direct, real-time feedback to the worker. The emergence of IoT-based ELF-EMF monitoring further enhances this capability by integrating edge computing and cloud-based analytics, which allow for real-time anomaly detection and longitudinal exposure forecasting [21]. Despite their promise, such devices face battery limitations, thermal drift, and a narrower dynamic range (~±100 µT) compared to some industrial settings which may result in underrepresentation of transient field peaks.

FBG-based systems, previously described, remain uniquely suited for EMI-heavy infrastructure settings. However, their deployment requires sophisticated calibration, temperature compensation, and higher initial setup costs, limiting their accessibility for routine monitoring. This contextual alignment is summarized in Table 11.

The preferred environment and best practices for categorization in Table 10 are grounded in case studies. EMDEX Lite was deployed for occupational shift monitoring [59], IoT meters supported smart grid surveillance [21], FBG sensors were used in substations [65], and SQUIDs proved valuable in neuroimaging labs [54].

### 6.3. Measurement Strategy for Biological Relevance

One of the critical limitations in both short- and long-term instruments is the disconnect between physical field measurements and physiological or biological outcomes. While short-term, high-sensitivity devices like SQUIDs enable localized correlation with brain or cardiac activity [23], wearable long-term sensors still lack integrated biosensing capabilities. Bridging this gap will require the development of multi-modal systems that can record both field intensity and biological markers such as heart rate variability or melatonin levels in real time [29,36]. Additionally, from a regulatory perspective, the IEE, IEEE, and ICNIRP emphasize the importance of duration-weighted exposure assessment, which favors long-term monitoring protocols that capture cumulative dose and exposure variability over time [1,2]. Spot measurements, while valuable for compliance verification, may misrepresent exposure risks if not accompanied by time-resolved contextual data.

### 6.4. Interpretive Limitations and Calibration Challenges

Both approaches suffer from calibration complexity and performance degradation over time. Sensor drift, temperature dependence, and environmental noise affect all instruments to varying degrees and require routine recalibration using traceable field generation systems such as Helmholtz coils [70]. Short-term measurement devices are particularly vulnerable to spatial inconsistency in heterogeneous field environments, while long-term systems must mitigate power consumption and sampling limitations that may compromise data fidelity [65,100].

### 6.5. Comparative Argument: Towards a Hybridized Monitoring Model

Considering the strengths and weaknesses of each approach, a single instrument or measurement strategy is unlikely to be universally applicable. However, for the majority of real-life exposure scenarios, particularly those requiring cumulative exposure data, regulatory compliance, or risk modeling, long-term monitoring emerges as the more robust and informative approach. The ability to continuously capture fluctuating field intensity, correlate exposure with physiological outcomes, and support predictive analytics makes long-term instruments better aligned with modern public health and occupational safety goals. That said, short-term instruments remain indispensable for high-resolution diagnostics, rapid assessments, and experimental exposure characterization, particularly in laboratory and clinical research contexts.

Several strategic recommendations can be made to improve ELF-EMF measurement practices and support the development of more accurate standardized biologically meaningful monitoring frameworks. Long-term monitoring instruments should be prioritized in occupational, residential, and environmental studies due to their ability to compare cumulative and time-varying ELF-EMF exposure. Short-term tools remain useful in laboratory and diagnostic contexts but are limited in dynamic field applications. Future systems should integrate biosensors to link exposure data with physiological responses, enabling more meaningful health assessments. Standardized calibration protocols, aligned with ICNIRP and IEEE guidelines, are essential to ensure measurement reliability. Continued innovation will be critical to improving measurement accuracy, biological relevance, and regulatory utility.

### 6.6. Comparative Strengths and Weaknesses in Practical Application

The previous sections distinguished short-term and long-term ELF-EMF measurement techniques; this section, however, consolidates their respective strengths and limitations through a practical, application-driven lens reinforced by real-world case studies.

Short-term instruments offer high-resolution measurements and remain essential in clinical diagnostics. These instruments are, however, constrained by their dependence on cryogenic cooling, electromagnetic shielding, and an inability to record cumulative exposure over time—factors that limit their use in real-world or long-term field monitoring settings [54]. Long-term instruments, such as wearable dosimeters, fiber-optic magnetometers, and IoT-enabled ELF-EMF meters, offer continuous data capture and temporal averaging, supporting exposure modeling and epidemiological assessment. In a landmark field study, Valič et al. [59] utilized EMDEX Lite wearable dosimeters among utility workers, identifying peak exposure intervals during work shifts and enabling more accurate estimation of time-weighted averages (TWAs), a critical metric in occupational health evaluations. Similarly, Tampouratzis et al. [21] deployed IoT-enabled ELF-EMF sensors across smart grid environments, demonstrating real-time remote exposure surveillance and anomaly detection, which is essential for infrastructure risk monitoring. Nonetheless, long-term systems pose challenges such as calibration drift, power constraints, and narrower dynamic ranges (typically ± 100 µT), which may underrepresent transient field peaks. Fiber-optic systems, such as those deployed by Sousa et al. [65] in high-voltage substations, are valued for their EMI immunity and spatial fidelity but require advanced calibration and incur higher implementation costs. To clarify the practical distinctions between short-term and long-term ELF-EMF measurement approaches, Table 12 below summarizes their comparative strengths, limitations, and real-world applications.

This comparative summary reinforces the practical distinctions outlined above and directly supports the instrumentation framework proposed in this review.

### 6.7. Compliance with ICNIRP and IEEE Standards in Practical Measurement

While this review references international standards such as those from the International Commission on Non-Ionizing Radiation (ICNIRP) and IEEE C95.6-2002, a more explicit discussion is warranted to clarify how existing ELF-EMF measurement instruments align with their technical and procedural requirements. Both the ICNIRP and IEEE prescribe frequency-specific exposure limits, 200 µT for the general public and 100 µT for occupational exposure at 50 Hz, and require that measurements reflect root mean square(RMS) field values averaged over defined intervals, typically six minutes for public environments and up to thirty minutes in occupational contexts. Ensuring compliance involves several operational parameters. Instruments must be calibrated against traceable field sources such as Helmholtz coils to maintain measurement uncertainty within the ±3 dB tolerance typically required for regulatory validity. Devices must be capable of three-axis magnetic field measurement and must record time-averaged RMS values rather than instantaneous peaks, especially in environments where field strengths fluctuate. For instance, wearable dosimeters like EMDEX Lite, as used by Valič et al. [59], comply with these protocols by integrating continuous sampling and automated temporal averaging aligned with occupational guidelines. Similarly, IoT-based ELF meters such as those deployed by Tampouratiz et al. [21] support remote data logging and periodic calibration, consistent with IEEE’s guidance on long-term monitoring infrastructures. Measurement setup must also account for spatial orientation and waveform complexity. The ICNIRP emphasizes that non-sinusoidal waveforms, common in industrial environments, can increase biological interaction potential, necessitating spectral decomposition or harmonic analysis where applicable. In this regard, advanced devices equipped with FFT capabilities or waveform logging functions offer greater regulatory alignment. For environments with high electromagnetic interference, instruments such as fiber-optic Bragg grating (FBG) systems demonstrated by Sousa et al. [65] are preferred as they maintain accuracy without coping. Although many of the instruments reviewed meet sensitivity and deployment requirements, standard compliance also depends on adherence to sampling protocols, calibration procedures, and consistent reporting. Future studies should explicitly document these parameters in accordance with ICNIRP and IEEE guidelines to improve scientific reliability and exposure data.

Despite its crucial role in ensuring data validity and regulatory compliance, standardization in ELF-EMF measurement remains underdeveloped. While the ICNIRP and IEEE provide foundational guidelines such as RMS averaging, tri-axial sampling, and calibration via Helmholtz coils, real-world implementation is fragmented, leading to inconsistencies in exposure metrics, temporal resolution, and waveform characterization. These gaps compromise comparability across studies and weaken the interpretability of exposure-health associations. Moreover, emerging technologies like wearable dosimeters and IoT-based ELF-EMF meters increasingly exceed the capabilities anticipated by existing standards, particularly in capturing biologically relevant parameters such as harmonic content and exposure variability [21,29]. To advance both scientific rigor and public health protection, there is an urgent need for harmonized, application-specific standards that incorporate frequency-domain analysis, biosensor integration, and unified protocols across all monitoring contexts.

### 6.8. Evaluation of the Decision-Making Framework for ELF-EMF Instrumentation

The decision-making framework presented in Section 5.4 provides a structured, context-sensitive method for selecting ELF-EMF measurement instruments. It was developed in response to challenges in the field, including inconsistent instrument use, variable data quality, and poor alignment between measurement methods and biological or regulatory goals. These limitations have been noted in both epidemiological reviews and technical field studies, where discrepancies in exposure data often stem from differences in device selection and deployment strategy [8,59]. The framework’s primary strength is its customizability. Users can assign weights to criteria such as measurement duration, sensitivity, environmental adaptability, and cost, allowing tailored decision-making across various settings, whether SQUID systems for laboratory-grade neuroimaging or wearable dosimeters for occupational monitoring. This flexibility supports use cases ranging from regulatory compliance to longitudinal health studies. In addition, the framework contributes to methodological harmonization by standardizing the evaluation of instrumentation. This is particularly valuable given the lack of consensus on how best to align field measurement practices with exposure guidelines from the ICNIRP and IEEE, which specify performance thresholds but offer limited operational guidance. By translating these abstract standards into actionable device selection, the framework bridges a critical implementation gap. Limitations of the current version include some dependence on expert scoring and the assumption of static device capabilities. While scores are derived from the empirical literature and technical reports, some subjectivity remains, particularly for emerging technologies with limited deployment data. Furthermore, the framework does not yet account for modular upgrades, interoperability, or integration with biosensor capabilities, which are increasingly relevant in AI-enhanced and health tracking applications. Despite these constraints, the framework provides a transparent, reproducible, and scalable approach to instrument selection. It facilitates evidence-based decisions, reduces exposure misclassification, and enhances study comparability. Its use could improve the interpretability of exposure–health associations, especially in settings where methodological variability hampers causal inference. This framework represents a practical advancement in ELF-EMF instrumentation strategy. With continued refinement, including modular assessment features and biosensor integration, it has strong potential to support both scientific inquiry and regulatory enforcement in a field where measurement precision and biological relevance are paramount.

## 7. Conclusions

This review highlights the strengths and limitations of short-term and long-term ELF measurement instruments across residential, occupational, biomedical, and infrastructure contexts. Long-term tools such as wearable dosimeters, IoT meters, and fiber-optic sensors are better suited for real-world, time-varying exposure assessment. In contrast, short-term instruments like Gauss meters and SQUIDs offer sensitivity but are best used for diagnostics or lab research due to limited temporal resolution. Instrument selection should be based on monitoring duration, sensitivity needs, and deployment context. While wearables and IoT systems enable continuous exposure tracking aligned with health guidelines, calibration stability, power requirements, and data fidelity remain challenges. Similarly, short-term tools excel in spatial precision but are constrained by environmental sensitivity and operational complexity. To ensure biologically meaningful and standardized monitoring, future systems should integrate biosensor capabilities and adhere to harmonized calibration and reporting protocols. This framework supports the evidence-based selection of ELF-EMF instruments for researchers, health professionals, and regulatory stakeholders. Despite advancements, practical deployment challenges remain underexplored. Future work should assess the interoperability of different ELF-EMF monitoring systems, standard maintenance and calibration protocols, and the availability and scalability of these instruments in low-resource or developing settings, warranting attention to ensure monitoring capabilities globally.

## Figures and Tables

**Table 1:** Comparative evaluation of ELF-EMF measurement instruments by operating principle, sensitivity, application suitability, and technical limitations.

Instrument Type	Operating Principle	Measured Field Range *	Advantages	Limitations	Ideal Use Case	Sources
Hall/TMR Gauss Meter	Lorenzo voltage via Hall/TMR sensors	~0.1–100 µT	Portable, low-cost, real-time	EMI-prone, spatially limited	Residential audits, spot checks	[71,72,73,74,75,76]
SQUID Magnetometer	Superconducting quantum interference	~1 pT–1 µT †	Ultra-high sensitivity, biomedical utility	Requires shielding, cryogenics, and a costly setup	MEG, neurophysiology labs	[54,77,78,79]
Wearable ELF Dosimeters	TMR with MCU and motion correction	0.01–100 µT [80]	Continuous logging, epidemiological profiling	Limited battery and range	Occupational monitoring, exposure modeling	[58,80,81,82]
IoT ELF-EMF Sensors	Magnetoresistive + wireless	~0.1–100 µT	Cloud analytics, shift data logging	Power/cost, limited high-current range	Smart grid, public infrastructure	[21]
Fiber-Optic (FBG) Sensors	Bragg reflection strain shift	~1 nT–1 mT †	EMI immunity, remote multi-point sensing	Temperature drift, complex calibration	HV substations, MRI zones, long-distance monitoring	[83,84,85,86]

* Values represent typical measurement ranges under operational conditions; minimum detectable field (sensitivity) may be lower. † SQUIDs and FBG sensors may detect much lower fields (e.g., femto/picotesla) under laboratory conditions, but listed ranges reflect real-world ranges.

**Table 2:** Quantitative performance metrics of ELF-EMF measurement instruments.

Instrument Type	Sensitivity	Accuracy	Sampling Rate	Power	Estimated Cost (USD)	Measurement Accuracy	Best Use	Sources
Hall/TMR Gauss Meters	0.1–100 µT	±2–5%	1–10 Hz	Battery (4–12 h)	USD 200–2000	±5–10%	Spot checks, residential audits	[72,76,87,88,89,90,91]
SQUID Magnetometers	1 fT–1 µT	±1–2% (lab)	10–1000 Hz	Cryogenic cooling	USD 50,000–200,000	±1–10%	Biomedical labs, MEG/neuroimaging	[92,93]
Wearable ELF Dosimeters	0.01–100 µT	±10%	0.1–1 Hz (average logging)	Rechargeable (8–24 h)	USD 1000–5000+	±10–15%	Occupational monitoring, time-averaged TWA	[57,58,94,95]
IoT Sensors	0.01–100 µT	±5–10%	1–5 Hz	Mains/Solar + backup	USD 500–3000	±10–15%	Smart grids, infrastructure surveillance	[72,96]
Fiber-Optic FBG Sensors	~1 nT–mT	±5%	1–10 Hz	Optical + signal processor	USD 5000–20,000+	±3–10%	EMI-Pro zones	[92,97]

**Table 3:** Summary of ELF-EMF measurement studies by instrument, context, and monitoring strategy.

Source	Instrument Type	Context	Measured Field Range	Strengths	Limitations	Technique Duration
[59]	Wearable TMR (tunneling magnetoresistive) Dosimeter	Occupational (field use)	~0.01–100 µT	Motion compensation, real-time logging	Limited dynamic range, battery-dependent	Long-term
[54]	High-Tc SQUID	Biomedical labs	~1 pT–1 µT	Ultra-high sensitivity, sub-picotesla detection	Requires cryogenic cooling, magnetic shielding	Short-term
[66]	Φ-OTDR FBG Sensor	Infrastructure monitoring	~0.1 nT–1 mT	EMI immunity, distributed sensing over a km range	Temperature cross-sensitivity, complex calibration	Short-term
[98]	MEMS-based Hall-effect Gauss Meter	Residential/Indoor	~0.1–100 µT	Portable, cost-effective, sub-nanotesla sensitivity	Susceptible to EMI, point measurement only	Short-term
[21]	IoT-based ELF Flux Density Meter	Industrial/Smart Grid	0.1–100 µT	Wireless data transfer, real-time analytics	Needs periodic calibration, limited by power supply	Long-term
[15]	Low-Tc SQUID (FLL)	Controlled lab studies	~1 pT–1 µT	Broad dynamic range, reduced drift via feedback	High cost, bulky setup	Short-term
[65]	FBG with Terfenol-D	HV substations, MRI rooms	~0.01–100 µT	Non-metallic, EMI-immune, remote sensing	Requires dual FBGs for temperature compensation	Long-term

**Table 4:** Evaluation criteria and default weights.

Criterion	Description	Suggested Weight (%)
Monitoring Duration Suitability	Ability of the instrument to support either short-term spot assessment or long-term continuous monitoring	20%
Sensitivity and Accuracy	Precision in biologically relevant ranges	20%
Environmental Adaptability	Operational stability in EMI-prone or mobile settings	15%
Biological/Regulatory Relevance	Alignment with ICNIRP/IEEE standards and health linkage	20%
Usability and Maintenance	Ease of operation, calibration needs, user burden, battery life, and mobility	15%
Cost and Scalability	Financial feasibility for individual or large-scale deployment	10%

**Table 5:** Instrument scoring matrix.

Instrument Type	Duration	Sensitivity	Env. Adaptability	Bio/Reg. Relevance	Usability	Cost
Wearable ELF Dosimeter (e.g., EMDEX Lite)	5	3	4	4	4	4
IoT-Enabled Sensor	5	3	4	4	3	3
Gauss meter (Hall/TMR)	2	3	2	2	5	5
SQUID Magnetometer	2	5	1	5	1	1
Fiber-Optic FBG Sensor	4	5	5	4	2	2

**Table 6:** Final weighted scores.

Instrument	Weighted Total (Out of 5)
Wearable ELF Dosimeter	4.05
IoT-Enabled ELF Sensor	3.75
Fiber-Optic FBG Sensor	3.55
Gauss meter	3.20
SQUID Magnetometer	2.70

**Table 7:** Adjusted weights for each criterion.

Criterion	Adjusted Weight (%)
Monitoring Duration	25
Sensitivity and Accuracy	15
Environmental Adaptability	20
Biological/Regulatory Relevance	15
Usability and Maintenance	15
Cost and Scalability	10

**Table 8:** Scoring of instruments for specific case use.

Instrument Type	Duration	Sensitivity	Env. Adaptability	Bio/Reg. Relevance	Usability	Cost
Wearable ELF Dosimeter (e.g., EMDEX Lite)	5	4	4	5	4	4
IoT-Enabled Sensor	5	3	4	4	3	3

**Table 9:** Weighted score for desired instruments.

Instrument	Weighted Total (Out of 5)
Wearable ELF Dosimeter	4.45
IoT-Enabled ELF Sensor	3.90

**Table 10:** Comparative evaluation of short-term vs. long-term ELF-EMF measurement approaches.

Parameter	Short-Term Instruments	Long-Term Instruments
Duration	Seconds to a few hours	Hours to days or longer [59]
Common Devices	Gauss meters, SQUIDS [54]	Wearables (EMDEX), FBG sensors, IoT meters [65]
Sensitivity	High (fT to nT range)	Moderate to high (µT to pT range) [66]
Strengths	High spatial resolution, rapid deployment, ideal for diagnostics	Continuous data, time-averaged, better for epidemiological and workplace tracking [21]
Limitations	No temporal exposure profile, subject to spot errors [99]	Battery/power constraints, thermal driving, requires calibration [100]
Best Use Case	Biomedical labs, compliance spot checks	Occupational studies, infrastructure monitoring, and public health surveillance
Biological Relevance	High (in lab/clinical studies) [57]	Moderate (real-world application, needs biosensor integration) [30]

**Table 11:** Contextual suitability of instrument types by environment and objective.

Instrument Type	Preferred Environment	Best For	Limitations
Hall/TMR Gauss Meters	Residential, low-EMI indoors	Spot measurements, public awareness	EMI-sensitive, limited spatial context [20]
SQUIDs (Low/High-Tc)	Biomedical labs, shielded rooms	Neurological and cardiological signal detection	High cost, low portability [54]
Wearable Sensors	Industrial, occupational	Longitudinal exposure tracking	Battery limits, limited dynamic range [59]
FBG Fiber-Optic Sensors	Substations, high EMI zones	Infrastructure monitoring, EMI immunity	Complex calibration, cost [65]
IoT-based ELF Meters	Smart grids, urban surveys	Remote tracking, real-time alerts	Power constraints, data reliability in dense EMI [21]

**Table 12:** Comparative summary of short-term **vs.** long-term measurement techniques.

Aspect	Short-Term Measurement	Long-Term Measurement
Typical instruments	Gauss meters, SQUID magnetometers	Wearable sensors (EMDEX Lite) IoT meters, FBG fiber sensors
Measurement duration	Seconds to hours	Hours to days/weeks
Main advantages	– High spatial resolution – Ideal for quick audits – Ultra-sensitive (e.g., SQUIDs)	– Captures temporal variation – Time-averaged data – Suitable for real-world exposure tracking
Main limitations	– Misses long-term exposure – Affected by ambient noise – Requires shielding (SQUID)	– Battery/power constraints – Requires calibration – Higher setup cost (e.g., FBG)
Best use cases	Compliance checks, lab diagnostics, and neuroimaging	Occupational exposure, infrastructure monitoring, epidemiology
Representative case study	– SQUIDs in biomedical MEG labs [54]	– Wearable EMDEX in utility workers [59] – IoT meters in smart grid [21]
Biological relevance	High (in lab settings)	High (in real-world studies when paired with health data)

## Data Availability

Not applicable.
